# Infiltration of CXCL9+ macrophages confers a favorable prognosis in breast cancer: Insights from an integrated single-cell RNA and bulk RNA sequencing study

**DOI:** 10.1371/journal.pone.0337175

**Published:** 2025-12-01

**Authors:** Bin Liang, Zhicai Duan, Siyu Long, Peng Zhou

**Affiliations:** 1 Department of General Surgery (Breast Surgery), The Affiliated Hospital of Southwest Medical University, Luzhou, People’s Republic of China; 2 Department of Ultrasound, The Affiliated Hospital of Southwest Medical University, Luzhou, People’s Republic of China; Sichuan University, CHINA

## Abstract

**Background:**

Breast cancer is the most common cancer among women and a leading contributor to disease-related mortality. While advancements in diagnostic techniques and the widespread dissemination of medical knowledge have improved outcomes, immunotherapy targeting the tumor microenvironment offers a promising approach to further reducing the disease burden.

**Methods:**

We first explored cellular heterogeneity within breast cancer tumors using single-cell RNA sequencing and subsequently analyzed the association between the infiltration levels of various cell types and survival prognosis using a deconvolution algorithm combined with bulk RNA sequencing. Our analysis identified CXCL9 + macrophages as being associated with improved survival outcomes. Next, WGCNA was used to identify genes related to CXCL9 + macrophage infiltration, and a survival risk model was constructed based on these genes. Finally, experiments were conducted to validate the effects of CXCL9 + macrophages on breast cancer cells.

**Results:**

We found that CXCL9 + macrophage primarily interacted with cytokines and activated T and NK cells through the CXCL9/10/11-CXCR3 axis. Increased infiltration, as observed in bulk RNA sequencing, was associated with improved patient survival. The risk model constructed based on CXCL9 + macrophage infiltration-related genes demonstrated strong efficacy in predicting patient survival outcomes. Finally, in vitro experiments confirmed that CXCL9 + macrophages inhibited the viability of breast cancer cells.

**Conclusion:**

CXCL9 + macrophages could play a significant role in inhibiting breast cancer, and their infiltration into tumor tissues is associated with improved survival in breast cancer patients. Immunotherapy targeting CXCL9 + macrophages hold great potential as a therapeutic strategy.

## Introduction

Breast cancer (BC) is the most common malignancy in women and remains the leading cause of cancer-related mortality [1,2]. Advances in medical knowledge, cancer screening, and treatment have improved patient survival [3]. However, advanced, and refractory recurrent breast cancers continue to pose significant challenges, necessitating the development of new therapeutic strategies.

Breast cancer is a highly complex and heterogeneous malignancy with a diverse genetic spectrum and classification [4]. Although various treatment modalities, including chemotherapy, radiotherapy, targeted therapy, hormone therapy, and surgical resection, are available, they are often associated with serious side effects, recurrence risk, and off-target effects [5]. Therefore, exploring alternative treatment approaches and mechanisms is crucial. With an increasing understanding of the tumor microenvironment and tumor immunity, immune-based anti-tumor strategies offer a promising new direction for breast cancer treatment [6].

Immunotherapy is closely linked to the exploration of the tumor microenvironment, which consists of various cells, cytokines, and the local physical and chemical environment [7]. These components profoundly influence tumor transformation, proliferation, metastasis, recurrence, and resistance to treatment [8]. Studies have shown that multiple immune cells contribute to breast cancer progression, including macrophages, T/NK cells [9–13].

For instance, M1 macrophages are generally associated with anti-tumor activity, whereas M2 macrophages suppress anti-tumor immunity. Additionally, the number of T cells varies among breast cancer subtypes, with triple-negative breast cancer (TNBC) exhibiting the highest levels, likely due to its relatively high tumor mutation burden [14]. In treated primary breast tumors, CD3 + T cells were abundantly infiltrated, particularly in Luminal B, HER2 + , and TNBC subtypes, with a high proportion of CD8 + T cells [15,16]. Furthermore, the ratio of CD8+ to CD4 + T cells increased with higher tumor-infiltrating lymphocyte (TIL) density, suggesting that elevated effector T cell levels in tumors with high TIL density correlate with improved clinical outcomes [11]. However, the relationship between more detailed immune profiles—particularly immune cell subpopulations linked to tumor suppression and progression—remains unclear.

Single-cell RNA sequencing offers a higher-resolution approach to exploring tumor heterogeneity and the interactions between cells [17], providing a deeper understanding of the role and relationships within the tumor microenvironment in breast cancer. However, while scRNA-seq analysis alone can dissect tumor heterogeneity at high resolution and characterize cellular features at single-cell resolution, it typically involves limited sample sizes and lacks corresponding prognostic information. Conversely, bulk RNA sequencing alone provides abundant survival and prognostic data from multi-patient cohorts but lacks cellular-level resolution. Therefore, integrating scRNA-seq with bulk RNA-seq combines the advantages of both approaches, enabling exploration of the relationship between immune infiltration and breast cancer and further elucidating patient prognosis. In this study, we utilized scRNA sequencing to analyze the characteristics and heterogeneity of immune-related cells in breast cancer. Combined with bulk RNA sequencing, we examined the impact of these immune-related cells on survival prognosis, identified key genes associated with immune cell infiltration, and proposed new insights for tumor treatment.

## Materials and methods

### Data acquisition and analysis

Data incorporated in this research comprised single-cell and bulk RNA sequencing, alongside clinical information. The single-cell RNA sequencing data encompass normal breast samples, HER2 + breast cancer, ER+ breast cancer, and triple-negative breast cancer, all obtained from the GEO database (GSE161529). Bulk RNA sequencing data for breast cancer, along with associated clinical data, were sourced from TCGA and XENA. Data processing and analysis were primarily conducted using R programming language.

### Single-cell RNA sequencing data analysis

scRNA sequencing data analysis was primarily performed using the R package Seurat. First, the data underwent quality control, where low-quality cells were filtered according to the following criteria: (1) Cells with fewer than 200 detected genes were excluded; (2) Genes expressed in fewer than three cells were excluded; (3) Cells with under 201 total gene expression counts (indicative of poor quality) or with over 8,000 detected genes (suggesting potential doublets) were removed; (4) Cells with mitochondrial gene-derived unique molecular identifiers (UMIs) exceeding 20% of the total were also excluded. Furthermore, mitochondrial, ribosomal, hemoglobin-related genes, as well as the MALAT1 gene, were removed from downstream analyses. Harmony was used to mitigate batch effects between different samples. For annotation, the FindAllMarkers function was employed to identify differentially expressed genes between clusters, and annotation was carried out based on existing knowledge and relevant literature.

### BayesPrism cell type deconvolution

We applied BayesPrism to perform cell type deconvolution on TCGA data using cell type annotations from single-cell sequencing data. This approach allowed us to calculate the proportion of different cell types in the bulk RNA sequencing data [18]. Cell type annotations from the single-cell RNA sequencing data were integrated with the bulk RNA-seq data as input for the analysis.

### Intercellular communication analysis

In this study, intercellular interactions between cell subpopulations were analyzed using CellPhoneDB [19]. CellPhoneDB estimates the potential interaction strength between two cell subpopulations based on the expression of ligand-receptor (LR) pairs across different cell types.

### Combined bulk-RNA seq analysis

Survival analysis was performed to investigate the relationship between the infiltration levels of various cell types in the bulk RNA sequencing data and patient survival outcomes, including overall survival (OS), disease-specific survival (DSS), progression-free interval (PFI), and disease-free interval (DFI). Weighted gene co-expression network analysis (WGCNA) was used to explore the relationship between cell type infiltration and associated gene modules. The PowerEstimate function was applied to determine the optimal soft threshold for identifying module-related genes associated with infiltration level traits for subsequent analysis. Cox regression and LASSO regression analyses were then conducted on the hub genes identified by WGCNA to further refine the selection of survival-related genes and construct risk scores.

### Risk score construction

To construct a prognostic risk model associated with CXCL9 + macrophage infiltration, we employed Weighted Gene Co-expression Network Analysis (WGCNA) to identify gene modules correlated with CXCL9 + macrophage infiltration levels. Genes from the identified modules were extracted for subsequent survival analysis. Least Absolute Shrinkage and Selection Operator (LASSO) Cox regression was performed to identify survival-related genes from the module genes. The LASSO model was implemented using the glmnet function with Cox family specification. Selected genes from LASSO regression were incorporated into a multivariate Cox proportional hazards model using the coxph function to evaluate their combined prognostic value. The risk score for each patient was calculated based on the expression levels of selected genes weighted by their respective regression coefficients derived from the multivariate Cox model using the predict function. To explore the functional relationships among the survival-related genes identified in our risk model, a protein-protein interaction (PPI) network was constructed to visualize the interconnections between these prognostic markers.

### Cell culture and transfection

MDA-MB-231 cells and the human monocytic leukemia cell line THP-1 were cultured in RPMI 1640 medium (Gibco, USA) supplemented with 10% fetal bovine serum (FBS, Gibco, USA). They were purchased from Pricella Biotechnology Co., Ltd. THP-1 cells were first induced into M0 macrophages using 100 ng/mL phorbol 12-myristate 13-acetate (PMA), and subsequently polarized into M1 macrophages with lipopolysaccharide (LPS). The conditioned medium from these macrophages was then used for co-culture experiments with MDA-MB-231 cells. All cells were maintained in a humidified incubator at 37°C with 5% CO₂. Small interfering RNA (siRNA) and transfection reagents were obtained from GenePharma.

### Reverse transcription quantitative PCR (RT-qPCR)

RNA extraction was performed using TRIzol (Invitrogen), and cDNA was generated with the Roche’s reverse transcription kit. The quantitative PCR assays were conducted using SYBR Green PCR Master Mix (Roche), following the manufacturer’s instructions. Primers were produced by Shanghai Sangon Biotechnology Co., Ltd.

### EdU assay

Cell proliferation was evaluated using an EdU incorporation assay kit (Abbkine, Wuhan, China). EdU was added to the culture medium according to the manufacturer’s instructions to label newly synthesized DNA during cell replication. EdU incorporation was subsequently detected using fluorescence microscopy.

### Transwell migration and invasion assays

Cell migration ability was assessed using a Transwell assay. A total of 10,000 cells were seeded into the upper chamber of a Transwell insert and incubated at 37°C with 5% CO₂ for 48 hours. For invasion assays, Transwell inserts were pre-coated with Matrigel and incubated at 37°C for 2 hours to allow gel polymerization before cell seeding. The same cell number (10,000 cells) was seeded and incubated under identical conditions. After incubation, migrated cells on the lower surface of the membrane were fixed with 4% formaldehyde and washed with phosphate-buffered saline (PBS). The cells were then stained with crystal violet solution. Excess stain was removed by rinsing with PBS, and non-migrated or non-invaded cells on the upper side of the membrane were gently wiped off with a cotton swab. Migrated and invaded cells were visualized and counted under a microscope.

### Colony formation assay

Approximately 500 cells were seeded into culture dishes and cultured for two weeks in conditioned medium collected from different macrophage cultures. Following incubation, colonies were fixed with 4% formaldehyde and stained with 0.1% crystal violet solution. The stained colonies were visualized and quantified using ImageJ software for image analysis.

### Statistical analysis and data visualization

Bioinformatics analyses were primarily conducted using R and Python. Survival analyses were performed using R software. Cox proportional hazards regression models were constructed using the coxph function to evaluate the association between immune infiltration scores and patient survival outcomes. Hazard ratios (HRs) were calculated to assess the prognostic significance of immune-related features. For Kaplan-Meier survival analysis, patients were stratified into high and low immune infiltration groups based on the median value of the immune infiltration score. Survival curves were generated using the survfit function. The survminer package was utilized for visualization and statistical analysis of survival curves through the ggsurvplot function. Log-rank tests were performed to determine statistical differences between the survival distributions of the high and low infiltration groups. GraphPad Prism 9 was used for data visualization and statistical analyses, while ImageJ and Fiji were employed for quantifying experimental images. Student’s t-test was used for comparisons between two groups, and one-way ANOVA was applied for multiple-group comparisons. Unless otherwise specified, statistical significance was defined as P < 0.05.

## Results

### Single-cell RNA sequencing reveals characteristics of breast cancer cell subpopulations

**Fig 1:**
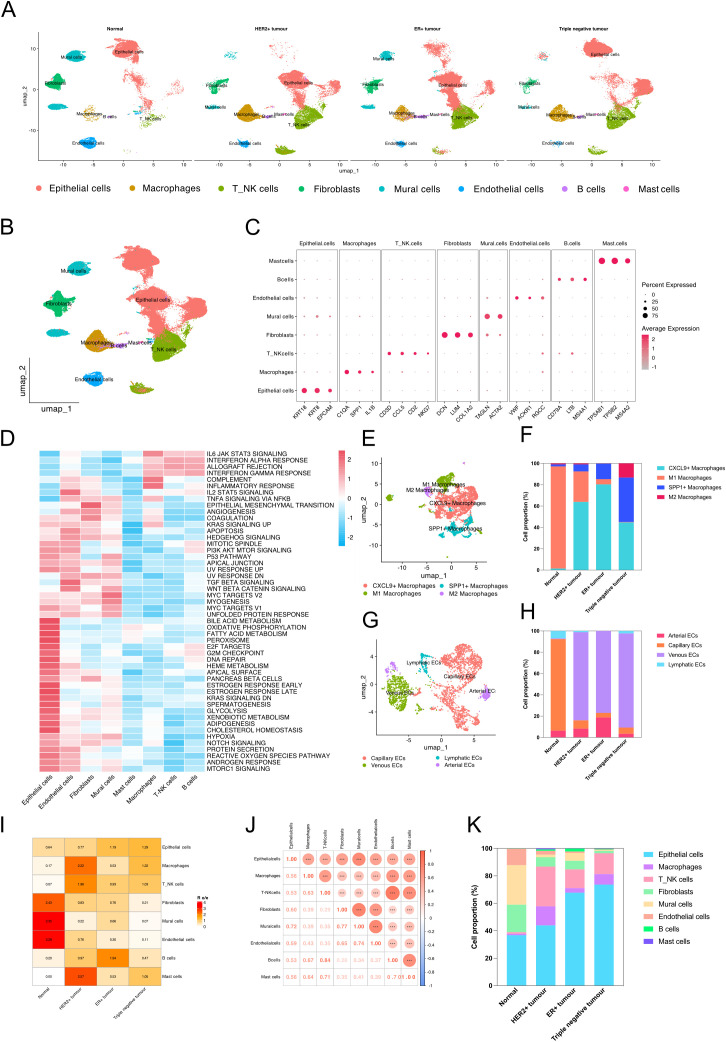
Cell clustering and annotation of breast cancer scRNA-seq data. **(A)** UMAP plot showing cell cluster annotations across normal breast tissue and breast cancer subtypes. **(B)** UMAP plot displaying annotations of all identified cell clusters. **(C)** Dotplot of specific marker genes for each cell subpopulation. **(D)** GSVA enrichment analysis of all clusters using HALLMARK gene sets. **(E)** UMAP plot showing subcluster annotation of macrophages. **(F)** Proportion of macrophage subpopulations across different sample groups. **(G)** UMAP plot showing subcluster annotation of endothelial cells. **(H)** Proportion of endothelial cell subpopulations across different sample groups. **(I)** Heatmap of the R o/e index across cell clusters to quantify tissue preference. **(J)** Correlation heatmap between cell subpopulations. **(K)** Cell composition proportions of all identified cell types across grouped samples.

Our study included single-cell sequencing data from normal breast samples, HER2 + breast cancer, ER+ breast cancer, and triple-negative breast cancer. After quality control, a total of 92,068 cells were included in the analysis (Fig 1A). We annotated all cells into eight subpopulations: epithelial cells, T/NK cells, macrophages, fibroblasts, endothelial cells, mural cells, B cells, and mast cells (Fig 1B). EPCAM was used as a specific marker to identify epithelial cells, which also highly express genes such as KRT18 and KRT8. T cells and NK cells were grouped together due to their similar expression profiles, with CD3D and NKG7 being the key markers. Macrophages were identified using markers like IL1B and SPP1, while fibroblasts predominantly expressed DCN and LUM. Endothelial cells were identified by the expression of genes such as VWF, RGCC, and AKCR1. ACTA2 served as a marker for mural cells, B cells expressed CD79A and LTB, and mast cells were identified by the expression of TPSAB1 and MS4A2 (Fig 1C).

Through GSVA enrichment analysis, we found that epithelial cells were significantly more active in pathways such as bile acid metabolism, oxidative phosphorylation, fatty acid metabolism, and cholesterol metabolism. Macrophages were notably active in the inflammatory response pathway (Fig 1D). We then annotated macrophages and endothelial cells into subgroups. In addition to distinguishing between M1 and M2 macrophages, we identified clusters with high expression of SPP1 and CXCL9, which were annotated as SPP1+ and CXCL9 + macrophages, respectively (Fig 1E). In normal samples, M1 macrophages comprised the largest proportion, while CXCL9 + macrophages predominated in HER2 + , ER + , and triple-negative breast cancer, accounting for 63.92%, 80.34%, and 44.69%, respectively. Notably, SPP1 + macrophages also represented a significant proportion in triple-negative breast cancer (Fig 1F). We further categorized endothelial cells into arterial ECs, capillary ECs, venous ECs, and lymphatic ECs (Fig 1G). Specific markers were used to distinguish these subtypes: SEMA3G for arterial ECs, RGCC for capillary ECs, VWF for venous ECs, and PDPN for lymphatic ECs. We observed that in normal samples, capillary ECs comprised the highest proportion, whereas in the other three types of breast cancer, venous ECs accounted for the largest proportion (Fig 1H).

To quantify the tissue preference of each subpopulation, we calculated the R o/e index. In normal samples, fibroblasts, mural cells, and endothelial cells exhibited higher R o/e values of 2.43, 3.35, and 3.28, respectively. In HER2 + breast cancer, mast cells had the highest R o/e value of 2.57. B cells showed the highest index of 1.94 in ER+ breast cancer, while in triple-negative breast cancer, macrophages and T/NK cells had higher indices of 1.22 and 1.03, respectively (Fig 1I). We also performed a correlation analysis between subpopulations and found a strong correlation between macrophages and mural cells, with a correlation coefficient of 0.77. Macrophages and T/NK cells, as well as B cells and mast cells, also displayed high correlations (Fig 1J). The cell proportion analysis revealed that epithelial cells were the predominant component in all samples, while mural cells accounted for a significant proportion in normal samples. In contrast, T/NK cells and macrophages had higher proportions in cancer tissues (Fig 1K).

### Proportion inference of scRNA-seq subpopulations in Bulk-RNA sequencing and analysis of their relationship with patient prognosis

**Fig 2:**
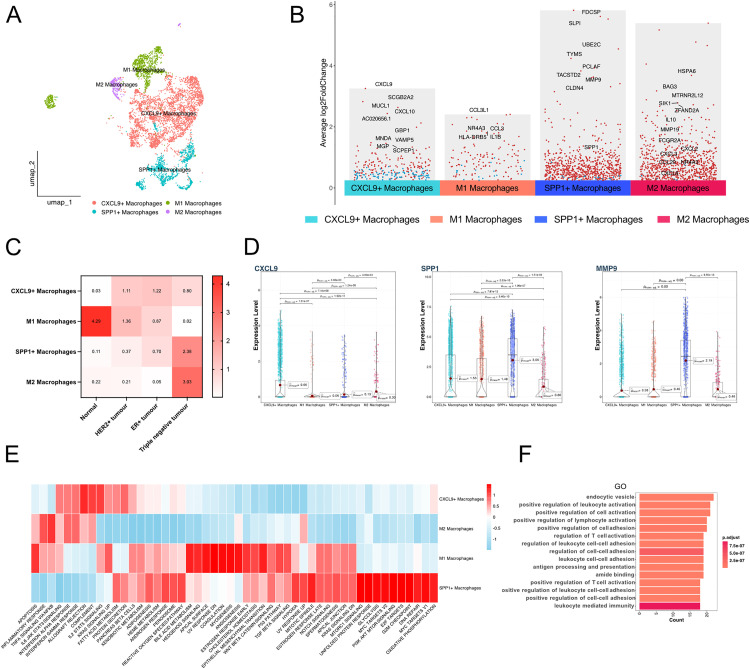
Inference of cell-type infiltration levels in bulk RNA-seq based on scRNA-seq annotations. **(A)** Comparison of infiltration proportions of all annotated cell types between normal and tumor samples in bulk RNA-seq data. **(B)** Correlation matrix showing the relationships among infiltration proportions of all cell types. **(C)** Forest plot summarizing Cox regression analysis of the association between cell infiltration proportions and breast cancer survival outcomes. **(D)** Kaplan–Meier (KM) survival curve for overall survival (OS), comparing high and low CXCL9 ⁺ macrophage infiltration groups divided by the median. **(E)** Volcano plot showing differentially expressed genes between high and low CXCL9 + macrophage infiltration groups. **(F)** KEGG pathway enrichment analysis of the differentially expressed genes. **(G)** Gene Ontology (Biological Process) enrichment analysis of the differentially expressed genes.

We applied the BayesPrism algorithm to calculate the proportion of subpopulations identified in single-cell RNA sequencing data within the bulk RNA sequencing data (Fig 2A). We observed that epithelial cells still constituted the majority, and mural cells represented a significant proportion in normal samples, consistent with the findings in the single-cell RNA sequencing data. A correlation analysis of subpopulation proportions in the bulk RNA sequencing data revealed high correlations between different types of macrophages, as well as between B cells and T/NK cells (Fig 2B).

Cox regression analysis was performed to assess the relationship between the infiltration proportions of each cell subpopulation and patient survival, with outcome indicators including overall survival (OS), disease-specific survival (DSS), disease-free interval (DFI), and progression-free interval (PFI). We found that the infiltration proportions of T/NK cells and CXCL9 + macrophages were associated with improved survival. Specifically, for T/NK cells: OS (HR: 0.75, P = 0.02), DSS (HR: 0.71, P = 0.05), DFI (HR: 0.80, P = 0.16), PFI (HR: 0.74, P = 0.02); and for CXCL9 + macrophages: OS (HR: 0.65, P = 0.004), DSS (HR: 0.56, P = 0.01), DFI (HR: 0.67, P = 0.04), PFI (HR: 0.73, P = 0.02) (Fig 2C), while other cell types, such as arterial ECs, showed no statistically significant association with survival.

Kaplan-Meier survival curve analysis further confirmed that CXCL9 + macrophage infiltration was beneficial to the survival of breast cancer patients (Fig 2D). In the bulk RNA sequencing data, we grouped samples based on the infiltration proportion of CXCL9 + macrophages for differential analysis. The high infiltration group showed significantly upregulated expression of genes such as CHIT1 (Fig 2E). KEGG pathway enrichment analysis revealed associations with the activation of pathways like cytokine-cytokine receptor interaction, chemokine signaling, and natural killer cell-mediated cytotoxicity (Fig 2F). Gene Ontology (GO) biological process (BP) pathway enrichment analysis highlighted associations with pathways such as leukocyte-mediated immunity, lymphocyte-mediated immunity, and the production of immune response molecular mediators (Fig 2G).

### Landscape of CXCL9 + macrophages at single-cell RNA sequencing resolution

**Fig 3:**
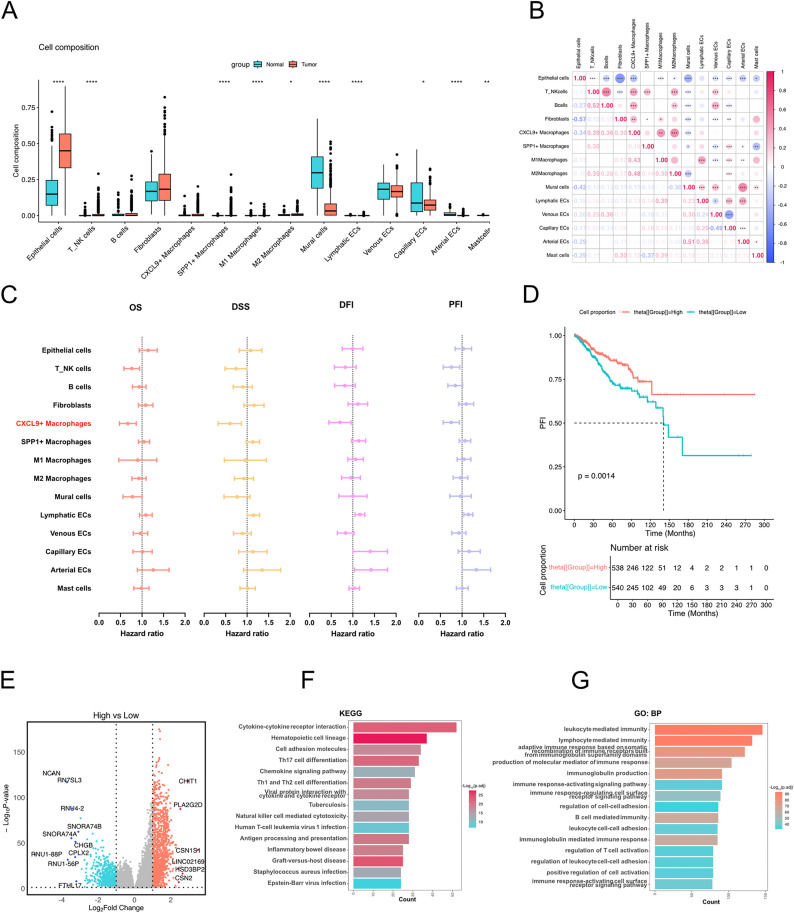
Single-cell transcriptional landscape of macrophage subpopulations. **(A)** UMAP plot showing the annotation of macrophage subpopulation. **(B)** Volcano plot displaying differentially expressed genes for different macrophage subpopulations. **(C)** Heat map of the R o/e index illustrating tissue preferences of macrophage subtypes. **(D)** Violin plots comparing the expression levels of CXCL9, SPP1, and MMP9 across macrophage subpopulation. **(E)** GSVA enrichment analysis of macrophage subpopulations using the HALLMARK gene set. **(F)** GO (Biological Process) enrichment analysis of highly expressed genes in CXCL9 + macrophages.

We further investigated the characteristics of CXCL9 + macrophages at the single-cell RNA sequencing level (Fig 3A). By analyzing the highly expressed genes of four macrophage subtypes, we found that in addition to highly expressing CXCL9, CXCL9 + macrophages also expressed key M1 macrophage markers such as CXCL10. In contrast, SPP1 + macrophages co-expressed SPP1 and MMP9. M1 macrophages predominantly expressed genes like IL1B and HLA-DRB5, while M2 macrophages were characterized by the expression of IL10, MMP19, FCGR2A, and CCL20 (Fig 3B). We confirmed through subgroup analysis that CXCL9 was mainly expressed in CXCL9 + macrophages, whereas SPP1 and MMP9 were predominantly expressed in SPP1 + macrophages (Fig 3D).

The R o/e index indicated that M1 macrophages exhibited a stronger preference in normal samples, with an R o/e index of 4.29, while CXCL9 + macrophages showed a preference across all three breast cancer types. Notably, in TNBC, SPP1 + macrophages and M2 macrophages had higher R o/e indexes of 2.38 and 3.03, respectively (Fig 3C). GSVA pathway enrichment analysis revealed that CXCL9 + macrophages were more active in pathways such as IL2-STAT5 signaling (Fig 3E). GO enrichment analysis of biological processes (BP) highlighted pathways primarily associated with endocytic vesicle formation, positive regulation of leukocyte activation, and positive regulation of cell activation (Fig 3F).

### Analysis of intercellular communication

**Fig 4:**
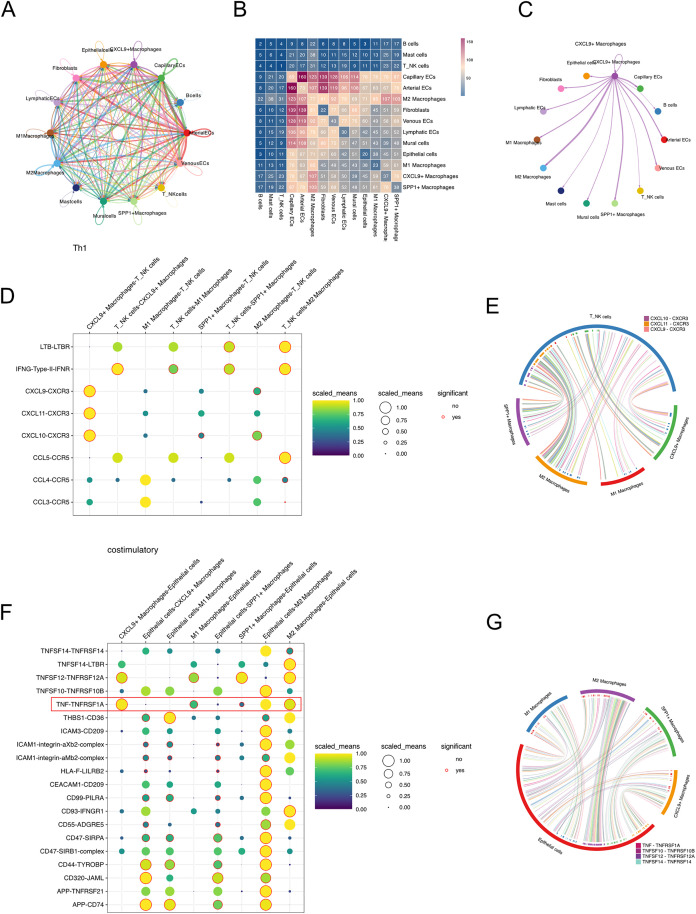
Cell-cell communication analysis among single-cell subpopulations. **(A)** Interaction intensity map among all annotated cell types. **(B)** Heatmap displaying the number of ligand–receptor interactions between cell populations. **(C)** Interaction intensity map showing communication between CXCL9 + macrophages and other cell types. **(D)** Dotplot of ligand–receptor interactions between macrophages and T_NK cells within the Th1-related gene family. **(E)** Chord diagram illustrating the interactions between macrophages and T_NK cells. **(F)** Dot plot of ligand–receptor interactions between macrophages and epithelial cells within the costimulatory gene family. **(G)** Chord diagram illustrating the interactions between macrophages and epithelial cells.

We utilized CellPhoneDB to investigate the intercellular communication between various subpopulations (Fig 4A). The heatmap analysis revealed that communication between Arterial ECs, Capillary ECs, Venous ECs, Fibroblasts, and M2 macrophages was relatively abundant. Additionally, CXCL9 + macrophages showed significant communication with Arterial ECs, Capillary ECs, M2 macrophages, and SPP1 + macrophages (Fig 4B, 4C). CXCL9 + macrophages predominantly interact with T/NK cells through the CXCL9-CXCR3, CXCL11-CXCR3, and CXCL10-CXCR3 signaling axes (Fig 4D, 4E). In interactions between macrophages and epithelial cells, CXCL9 + macrophages primarily communicate with epithelial cells via the TNF-TNFRSF1A pathway (Fig 4F, 4G).

### Construction of prognostic risk model based on genes related to CXCL9 + macrophage infiltration

**Fig 5:**
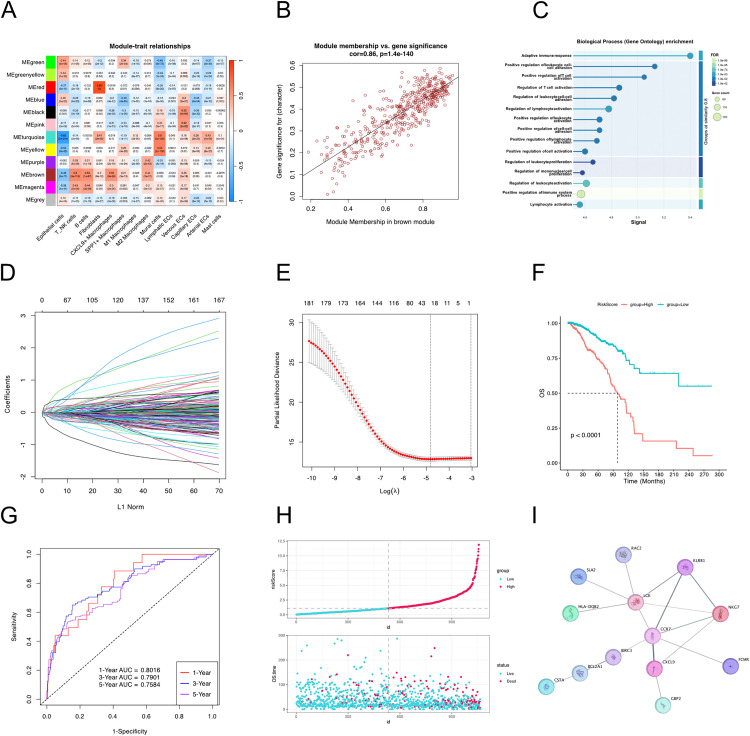
WGCNA analysis and construction of a prognostic risk score based on CXCL9 + macrophage-related genes. **(A)** Heatmap showing the correlation between WGCNA modules and cell infiltration traits. **(B)** Scatter plot illustrating the correlation between module membership and gene significance. **(C)** GO enrichment analysis of genes in the module most associated with CXCL9 + macrophage infiltration. **(D)** LASSO regression path plot for selecting survival-associated genes. **(E)** Ten-fold cross-validation results from LASSO regression showing the optimal lambda value. **(F)** Kaplan–Meier survival curve comparing high- and low-risk groups based on the median risk score. **(G)** ROC curves showing the predictive performance of the risk score for 1-year, 3-year, and 5-year survival. **(H)** Scatter plot showing the relationship between individual risk scores and survival status. **(I)** Gene interaction network of genes used to construct the risk score model.

We began by analyzing the modules associated with various cell infiltration levels using WGCNA (Fig 5A). The Brown module showed the highest correlation with CXCL9 + macrophage infiltration and also exhibited a strong correlation with T/NK cells. The correlation coefficient between module membership and gene significance was 0.86, indicating that the core genes in this module were highly correlated with traits of interest (Fig 5B). Pathway enrichment analysis of these genes revealed significant associations with immune regulation pathways, such as positive regulation of leukocyte cell-cell adhesion, positive regulation of T cell activation, and positive regulation of leukocyte activation (Fig 5C).

Next, we selected genes from this module to construct a survival-related risk score. Univariate Cox regression analysis was first applied to identify genes associated with overall survival (OS). The significant genes were further refined using LASSO regression to construct the final risk score (Fig 5D, 5E). Based on the median value of the risk score, patients were divided into high-risk and low-risk groups. Kaplan-Meier survival analysis revealed that the high-risk group had significantly worse prognosis (P < 0.0001) (Fig 5F, 5H). The ROC curve analysis based on the risk score demonstrated strong predictive performance, with AUC values of 0.80, 0.79, and 0.76 for 1-, 3-, and 5-year survival, respectively (Fig 5G). Finally, we constructed a gene interaction network for the genes included in the risk score. This network encompassed genes such as CCR7, CXCL9, GBP2, FCMR, NKG7, LCK, KLRB1, RAC2, SLA2, HLA-DQB2, BIRC3, BCL2A1, and CSTA, which were used to construct the prognostic risk score (Fig 5I).

### CXCL9 knockdown attenuates the inhibitory effect of M1 macrophages on breast cancer cells

**Fig 6:**
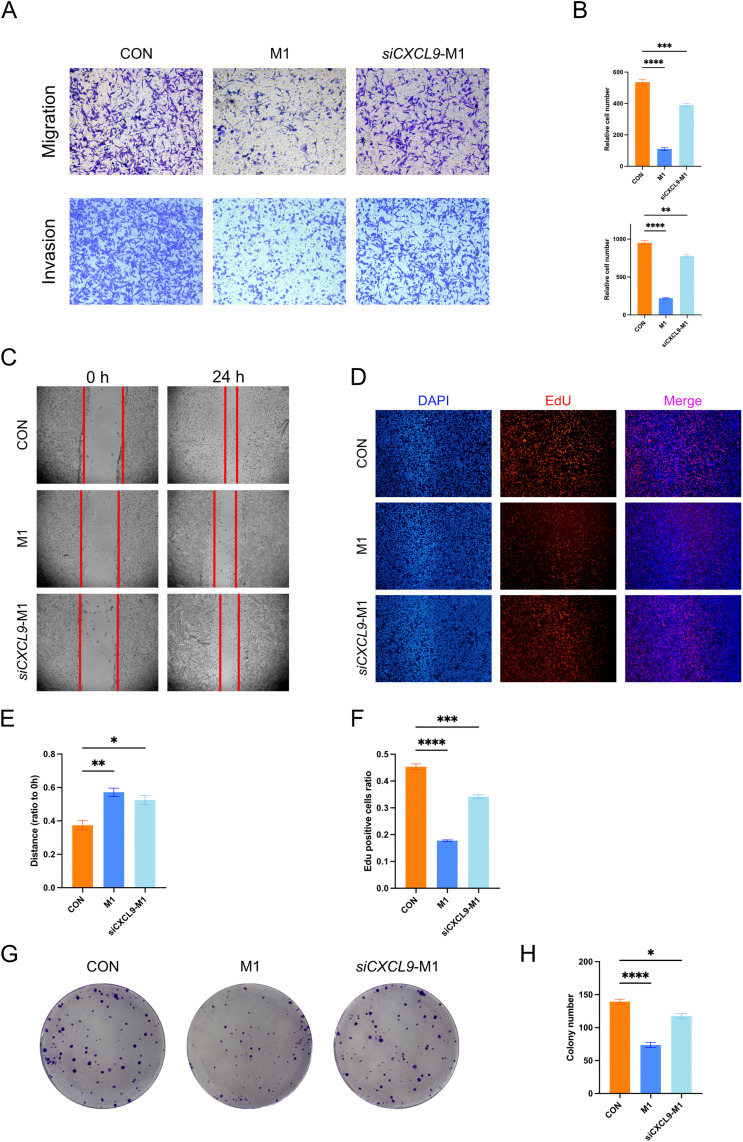
Effects of Macrophage-Conditioned Media on MDA-MB-231 Cells. **(A)** Transwell migration and invasion assay of MDA-MB-231 cells co-cultured with conditioned media from M0 macrophages (CON), M1 macrophages (M1), and siCXCL9 M1 macrophages (siCXCL9). **(B)** Quantification of migrated and invaded cells from the Transwell assay. **(C)** Wound healing assay showing migration of MDA-MB-231 cells at 0- and 24-hours post-scratch. **(D)** EdU incorporation assay to assess cell proliferation; nuclei were counterstained with DAPI. **(E)** Quantification of wound healing, expressed as the ratio of scratch distance at 24 hours to that at 0 hours. **(F)** Quantification of EdU assay, expressed as the percentage of EdU-positive cells. **(G)** Colony formation assay. **(H)** Quantification of colony numbers. Data are presented as mean ± SD. Statistical significance: ns, p ≥ 0.05; * p < 0.05; ** p < 0.01; *** p < 0.001; **** p < 0.0001.

To investigate the role of CXCL9 + macrophages in breast cancer, we conducted in vitro co-culture experiments using conditioned media from M0 macrophages, M1 macrophages, and siCXCL9 M1 macrophages, which were applied to MDA-MB-231 breast cancer cells. Transwell migration and invasion assays showed that the conditioned medium of M1 macrophages significantly inhibited the migration and invasion of MDA-MB-231 cells, and this inhibitory effect was significantly attenuated after knockdown of CXCL9 in M1 macrophages (Fig 6A, 6B). Similarly, wound healing assays demonstrated that CXCL9 knockdown attenuated the inhibitory impact of M1 macrophage-conditioned media on cell migration (Fig 6C, 6E). Furthermore, EdU incorporation assays showed that M1 macrophage-conditioned media significantly suppressed cell proliferation, while this suppression was weakened after CXCL9 knockdown (Fig 6D, 6F). Similarly, colony formation assays demonstrated that conditioned medium from M1 macrophages inhibited tumor cell colony formation, and this inhibitory effect was diminished when CXCL9 was knocked down in M1 macrophages (Fig 6G, 6H).

## Discussion

The tumor microenvironment (TME) plays a critical role in the development, progression, and treatment of breast cancer. Traditionally, research and treatment have focused on the cancer cells themselves, but this approach often leads to challenges such as drug resistance and treatment failure. The TME is a complex and dynamic ecosystem composed of tumor cells, immune cells, vascular endothelial cells, fibroblasts, and non-cellular components [6]. While the interactions between tumor cells and other cells in the TME can promote tumor progression, various cell types, including immune cells and endothelial cells, also play a crucial role in tumor suppression and can be targeted for therapeutic purposes. Therefore, the tumor microenvironment presents both a challenge and an opportunity: it can enhance the effectiveness of current treatments and pave the way for the development of entirely new strategies for treating and preventing breast cancer.

In this study, we explored the heterogeneity of various cell types across different breast cancer subtypes using single-cell RNA sequencing. We initially classified the cells into eight major subtypes: Epithelial cells, T_NK cells, Macrophages, Fibroblasts, Endothelial cells, Mural cells, B cells, and Mast cells. Further classification was performed for endothelial cells and macrophages. Endothelial cells were subdivided into Arterial ECs, Capillary ECs, Venous ECs, and Lymphatic ECs [20, 21]. Macrophages were not only categorized based on the conventional M1 and M2 phenotypes but were also further annotated into CXCL9 + macrophages and SPP1 + macrophages based on their high expression of CXCL9 and SPP1, respectively.

Ruben Bill et al. first proposed defining macrophage polarity using the CXCL9:SPP1 expression ratio, which is closely associated with the polarization of immune, stromal, and tumor cells within the tumor microenvironment [22]. We observed significant differences in cell infiltration between normal and cancerous tissues. In cancer samples, the proportion of T_NK cells and macrophages was notably higher, likely reflecting an immune response triggered by the tumor. Additionally, the Ro/e index revealed distinct tissue preferences among different cell types. Notably, macrophages and T_NK cells demonstrated higher Ro/e index in TNBC. Previous studies have shown that tumor-associated macrophages (TAMs) are extensively recruited in TNBC lesions and are associated with poor clinical outcomes [23].

We then calculated the infiltration proportion of cell subpopulations, as annotated by single-cell RNA sequencing, in Bulk-RNA sequencing data using BayesPrism deconvolution approach to explore its relationship with survival. The results of Cox regression analysis revealed that the infiltration level of CXCL9 + macrophages was associated with a reduced risk of poor outcomes when OS (HR: 0.65, P = 0.004), DSS (HR: 0.56, P = 0.01), DFI (HR: 0.67, P = 0.04), PFI (HR: 0.73, P = 0.02) were used as outcome indicators. Notably, T_NK cells also demonstrated a similar low-risk effect, although the association was not statistically significant for DSS (HR: 0.71, P = 0.05) and DFI (HR: 0.80, P = 0.16). This suggests that the infiltration of CXCL9 + macrophages and T_NK cells may be beneficial to breast cancer patients’ survival, with a potential reciprocal relationship between them [24]. T cell infiltration in the tumor microenvironment has emerged as a key determinant of immunotherapy efficacy and patient prognosis [25].

Furthermore, differential gene enrichment analysis, based on the infiltration of CXCL9 + macrophages in bulk-RNA data, showed that CXCL9 + macrophages were closely linked to cytokine signaling and immune regulation pathways. These findings suggest that CXCL9 + macrophages may inhibit tumor progression directly by interacting with tumor cells or by regulating other immune cells to modulate the immune response. This finding, along with previous results showing that infiltration of T/NK cells is associated with better prognosis in breast cancer patients, suggests that the infiltration of CXCL9 + macrophages may be related to T/NK cell activation, potentially involving the direct killing of tumor cells by activated T/NK cells.

At the single-cell level, we investigated differences among the four macrophage subtypes and their tissue preferences. Notably, SPP1 + macrophages and M2 macrophages exhibited higher R o/e indexes in triple-negative breast cancer (TNBC). M2 macrophages are generally associated with the suppression of anti-tumor immunity, promotion of tumor-associated angiogenesis, and facilitation of disease progression. Zhang et al. demonstrated that deubiquitination of YAP in macrophages promotes M2 phenotype polarization, contributing to TNBC progression [26]. GO enrichment analysis of highly expressed genes in CXCL9 + macrophages revealed that this subtype is associated with the positive regulation of leukocyte activation, suggesting a potential role in enhancing immune responses against tumors.

Our intercellular communication analysis identified the significant regulatory relationships and potential mechanisms of intercellular communication between CXCL9 + macrophages and other cell types, particularly between CXCL9 + macrophages and T/NK cells and epithelial cells, to explore the potential regulatory mechanisms by which CXCL9 + macrophages contribute to better prognosis in breast cancer patients. Cell-cell communication analysis revealed that CXCL9 + macrophages primarily regulate T_NK cells via the CXCL9-CXCR3, CXCL11-CXCR3, and CXCL10-CXCR3 axes. These signaling pathways play a crucial role in modulating immune cell migration, differentiation, and activation [27,28]. CXCR3 is highly expressed in Th1 cells, cytotoxic T lymphocytes (CTLs), NK cells, and natural killer T (NKT) cells [29]. CXCL9, CXCL10, and CXCL11 stimulate immune responses by promoting Th1 polarization and activation, leading to the production of IFN-γ, TNF-α, and IL-2 [30]. These cytokines, in turn, enhance anti-tumor immunity by activating CTLs, NK cells, NKT cells, and macrophages [31]. The CXCL9/10/11-CXCR3 axis is currently considered a promising target for drug development. Zhang et al. demonstrated that combining CXCL9 gene therapy with cisplatin enhances tumor reduction in colon and lung cancers while promoting CTL activation [32]. Regarding interactions with epithelial cells, we observed that CXCL9 + macrophages predominantly communicate with epithelial cells through TNF-TNFRSF1A signaling. TNF-α, initially identified in 1975 for its ability to induce tumor hemorrhagic necrosis, has long been recognized as a potential anticancer factor [33]. It is one of the key proinflammatory cytokines secreted by M1 macrophages, playing a role in both tumor progression and chronic inflammation [34]. However, emerging evidence suggests that TNF-α can exert dual effects in cancer, either promoting carcinogenesis or inhibiting tumor growth depending on the specific cellular context. In breast cancer, TNF-α is believed to have a context-dependent role, exerting both tumor-promoting and tumor-suppressing effects [35]. Notably, the interactions between macrophages and tumors are multifaceted. On one hand, macrophages directly or indirectly interact with other immune cells to influence tumor progression, as demonstrated by our cell-cell communication analysis, where the interactions between CXCL9 + macrophages and T cells/NK cells, particularly through the CXCL9-CXCR3, CXCL11-CXCR3, and CXCL10-CXCR3 axes, may substantially affect tumor progression. On the other hand, CXCL9 + macrophages can also directly interact with tumor cells, including releasing cytokines or remodeling the extracellular matrix environment.

We obtained the module genes associated with CXCL9 + macrophage infiltration through WGCNA and constructed risk score and interaction networks. Gene enrichment analysis within genes in the module also showed that CXCL9 + macrophage infiltration was associated with the activation of lymphocytes and T cells. Based on the analysis above, we believe that the favorable prognosis associated with CXCL9 + macrophage infiltration may be related to the direct regulation of T cells and NK cells. The activation of T/NK cells in the breast tissue warrants further investigation, especially within the immunosuppressive tumor microenvironment. This suggests that CXCL9 + macrophages could be a potential therapeutic target for activating anti-tumor immunity. Tumor microenvironments often exhibit immunosuppressive characteristics, hindering the activation and function of normal immune cells, thus compromising the body’s immune response against tumors [36]. However, this also presents potential opportunities for cancer treatment. In recent decades, researchers have continuously explored and unlocked the potential of immunotherapy in cancer treatment, including approaches such as immune checkpoint inhibitors, adoptive cell therapy, and cancer vaccines [37,38]. The role of macrophages, particularly their relationship with tumor progression, has also been the subject of increasing research [39,40]. By combining the high-resolution advantages of single-cell RNA sequencing for cell subpopulation analysis with the rich patient prognosis information from bulk RNA sequencing, we explored the potential anti-tumor capabilities of CXCL9 + macrophages and discussed their possible mechanisms. Our study provides a potential direction for future research. And the genes used to construct the risk model included CCR7, CXCL9, NKG7 and CSTA. NKG7, primarily expressed by NK cells, plays a crucial role in controlling tumor initiation, growth, and metastasis [41]. Cystatin A (CSTA), also known as stefin A, functions as an inhibitor of cysteine proteases and is recognized as a tumor suppressor. Loss of CSTA expression in breast tumors disrupts the balance of cysteine proteases, leading to enhanced extracellular matrix remodeling, tumor invasion, and metastasis [42]. Notably, multiple studies have demonstrated that CSTA expression correlates with improved prognosis in cancer [43–45]. In bulk RNA sequencing analysis, a risk score based on a gene signature comprising CXCL9 and T/NK cell-related genes showed good predictive power for breast cancer prognosis. Furthermore, the clustering of these genes in the WGCNA suggests a close relationship between CXCL9-related macrophages and T/NK cells, corroborating the findings from single-cell sequencing analysis. However, identifying these closely related genes solely through bulk RNA sequencing may not provide sufficient insight into the regulatory mechanisms by which macrophages and T/NK cell subpopulations collaborate to suppress tumor growth, even if their gene signature correlates with breast cancer patient outcomes.

Finally, our in vitro experiments demonstrated that the knockdown of CXCL9 in M1 macrophages attenuates their inhibitory effect on breast cancer cells. Both migration and wound healing assays demonstrated that the suppression of MDA-MB-231 cell migration by M1 macrophage-conditioned media was diminished following CXCL9 knockdown. Similarly, the EdU proliferation assay revealed a reduced inhibitory effect on cell proliferation. It is important to note, however, that the anti-tumor effects of CXCL9 + macrophages likely extend beyond direct interactions with tumor cells and may also involve indirect modulation of other immune cells, such as T cells, NK cells, and B cells. Thus, these findings represent only one aspect of their broader functional impact. Therefore, the anti-tumor effects of CXCL9 + macrophages are multifaceted, encompassing both their direct killing and inhibitory effects on epithelial cells, as well as their modulation of the immune microenvironment to suppress tumor growth through other immune cells.

In conclusion, by integrating single-cell RNA sequencing and bulk RNA-seq analysis, we demonstrate that CXCL9 + macrophages are associated with improved survival outcomes in breast cancer. These results offer novel insights into the tumor microenvironment and highlight CXCL9 + macrophages as potential targets for immunotherapeutic strategies in breast cancer.
